# Developing a fit-for-purpose composite symptom score as a symptom burden endpoint for clinical trials in patients with malignant pleural mesothelioma

**DOI:** 10.1038/s41598-024-62307-5

**Published:** 2024-06-27

**Authors:** Charles S. Cleeland, Karen N. Keating, Brian Cuffel, Cem Elbi, Jonathan M. Siegel, Christoph Gerlinger, Tara Symonds, Jeff A. Sloan, Amylou C. Dueck, Andrew Bottomley, Xin Shelley Wang, Loretta A. Williams, Tito R. Mendoza

**Affiliations:** 1 Department of Symptom Research, The University of Texas MD Anderson Cancer Center, Houston, TX USA; 2 Bayer HealthCare Pharmaceuticals, Whippany, NJ USA; 3 Bayer AG, Berlin, Germany; 4 Gynecology, Obstetrics and Reproductive Medicine, University Medical School of Saarland, Homburg/Saar, Germany; 5 Clinical Outcomes Solutions, Folkestone, Kent UK; 6 Division of Biomedical Statistics and Informatics, Department of Health Sciences Research, Mayo Clinic, Rochester, MN USA; 7 Section of Biostatistics, Division of Health Sciences Research, Mayo Clinic, Phoenix, AZ USA; 8 Quality of Life Department, European Organisation for Research and Treatment of Cancer, Brussels, Belgium; 9 Present Address: Symptom Assessment Systems, 1416 Marconi St, Houston, TX USA; 10 Present Address: National Cancer Institute Office of Patient-Centered Outcomes, National Institutes of Health, Bethesda, MD 20814 USA; 11 Present Address: Bottomley Consulting Group, Brussels, Belgium

**Keywords:** Mesothelioma, Outcomes research

## Abstract

We developed a composite symptom score (CSS) representing disease-related symptom burden over time in patients with malignant pleural mesothelioma (MPM). Longitudinal data were collected from an open-label Phase IIB study in which 239 patients completed the validated MD Anderson Symptom Inventory for MPM (MDASI-MPM). A blinded, independent review committee of external patient-reported outcomes experts advised on MDASI-MPM symptoms to include in the CSS. Through iterative analyses of potential symptom-item combinations, 5 MPM symptoms (pain, fatigue, shortness of breath, muscle weakness, coughing) were selected. The CSS correlated strongly with the full MDASI-MPM symptom set (0.92–0.94) and the Lung Cancer Symptom Scale-Mesothelioma (0.79–0.87) at each co-administration of the scales. The CSS also had good sensitivity to worsening disease and global quality-of-life ratings. The MDASI-MPM CSS can be used as an outcome in MPM clinical trials, including in responder analyses and at the individual patient level. It is brief enough to administer frequently, including electronically, to better capture symptom trajectories during and after a trial and in clinical practice. As a single score, the CSS addresses multiplicity issues that can arise when several symptoms increase due to worsening disease. Our process can be adapted to produce a CSS for other advanced-cancer trials.

## Introduction

In oncology, stabilizing or reducing disease symptoms and improving functioning is inarguably beneficial for patients, especially those with late-stage disease. Agents that control disease progression could be expected to have beneficial effects on the various symptoms characteristic of that advanced disease. However, because each type of cancer could have a different set of associated symptoms, the severity of which will vary by disease stage, it is vital to distinguish disease-related symptoms from treatment-related symptomatic toxicities to the extent possible^1^.

Clinical trial endpoints that capture treatment benefits provide important information for multiple stakeholders—not only clinicians, patients, and caregivers, but also regulators, health technology assessment groups, and payers. If significant reduction or stabilization of disease-related symptoms, improvement of disease-related functional impairment, or decrease in treatment-induced toxicities compared with standard care or a competitor agent can be shown for a new therapy, these data could support regulatory agency approval and be possible candidates in a labeling claim or the supporting literature. Information on disease-related symptoms, symptomatic toxicities, functional impairment, and other aspects of health-related quality of life (QOL) can best be obtained from patient-reported outcomes (PROs)^1–4^.

It is well established that including PROs to capture the patient’s experience before, during, and after treatment is a vital component of the formal regulatory approval process for new therapies. In June 2021, the US Food and Drug Administration (FDA) issued draft guidance on the collection of core PROs in cancer clinical trials^4^. This draft guidance identified disease symptoms, symptomatic adverse events, and physical function as core outcomes of interest for FDA product review and presented characteristics necessary for a PRO tool to be considered “fit-for-purpose” for regulatory decision-making—meaning that the tool’s level of validation is sufficient to support its context of use^5^.

From a regulatory perspective, the following considerations are relevant in developing a PRO instrument: It must represent the target treatment group, must be enriched by qualitative research in the targeted population, must have acceptable psychometric properties, and must be able to capture expected clinical change; results must be easily interpretable by patients and clinicians; and the value that indicates a meaningful change in the score must have been examined^6^. Indeed, recent labels granted by the FDA show that PRO measures can be used to support labeling claims, so long as they are backed by evidence^1,7,8^.

Common symptoms experienced by patients with advanced cancer include pain, fatigue, muscle weakness, and lack of appetite^2^; however, at any given time, a patient might report only mild pain but distressingly high levels of other disease-related symptoms, such as shortness of breath or fatigue. It is problematic to analyze symptoms individually, because rating symptoms separately diminishes the statistical power to detect differences between groups. This contributes to sponsors’ concerns about using change in symptom burden as an endpoint in oncology clinical trials.

One solution is to develop a composite symptom score (CSS), sometimes called a total symptom score, comprising the key symptoms relevant to a given cancer and stage. These symptoms would be assessed individually in a clinical trial, but the CSS would be analyzed as a single variable that represents the construct of disease-related symptom burden. As a subset of the most salient and frequent symptoms reported by the targeted patient population, a CSS offers several advantages: (1) Inflation of alpha and loss of power caused by multiple comparisons of symptoms as individual outcomes are avoided, which might encourage the use of disease-related symptomatic impact as an outcome rather than as an exploratory endpoint^9^; (2) A CSS includes only a few items, significantly reducing patient assessment burden (requiring only 1–5 min to complete) and facilitating frequent electronic administration outside of the clinic, such that the trajectory of symptom status during and after completion of a clinical trial can be better captured; (3) A CSS could be used to represent disease-related symptom burden in responder analyses, which are encouraged by the FDA as a way to document change in PROs used as clinical outcome assessments^3^; and (4) A CSS would avoid the dilution effect that could occur if all symptoms of an instrument were included, because some disease-related symptoms will not be as prevalent or severe as others. Should between-group comparisons produce significant differences in the symptom burden represented by a CSS, additional analyses can be performed on the individual symptoms that contributed to the CSS. Composite symptom scores have been used successfully as endpoints in clinical trials for regulatory submissions^10,11^ and as secondary endpoints to compare treatments in other clinical studies^11^.

The aim of this study was to develop and evaluate a CSS in tandem with the validation of a new PRO measure, the MD Anderson Symptom Inventory for Malignant Pleural Mesothelioma (MDASI-MPM)^12^, for use as a potential disease-related symptom burden endpoint in pivotal trials. We describe how blinded, pooled data from a randomized Phase IIB clinical trial^13^ was used to develop the CSS, examine how this CSS performed longitudinally at baseline, during the trial, and at follow-up, and propose a value indicating a meaningful change in CSS score.

## Methods

This project is based on a collaboration between a pharmaceutical sponsor and an academic organization. The initial agreement included the development and validation of a fit-for-purpose PRO measure, the MDASI-MPM, that aligned with sponsor’s development of an agent designed to treat malignant pleural mesothelioma (MPM), an aggressive, highly symptomatic, and rapidly fatal cancer of the lung pleura. Patients with MPM report multiple severe symptoms that impair function and reduce treatment tolerance. After the qualitative and quantitative activities were completed for the MDASI-MPM, additional work was undertaken to develop a single CSS comprising a limited subgroup of the most relevant disease-related symptoms endorsed by patients with MPM as being easily understood and relevant to their stage of disease. As such, the CSS could be used to represent change in symptom burden for this patient population.

### MDASI-MPM development

The MDASI-MPM was developed from the core MD Anderson Symptom Inventory, a PRO instrument that measures 13 common cancer-related symptoms and 6 items describing symptom interference with functioning^14^. All symptom and interference items are rated on a 0–10 scale (from none to worst imaginable for symptom severity, and from none to completely interferes for interference items) with a recall period of the past 24 h. The 6 interference items can be divided into 2 subscales: a mood-related subscale comprising relations with others, enjoyment of life, and mood, and an activity-related subscale comprising ability to work, engage in daily activities, and walk. These 13 core symptoms and 6 interference items were derived from input from more than 500 patients with cancer of various types^14^ and also were debriefed and found relevant in the MPM patient sample^15^.

Disease-specific and treatment-specific symptom items can be added to the core MDASI’s 19 symptom severity and interference items to form new instruments specifically tailored to the disease or treatment of interest. A similar approach has been used with instruments such as the EORTC QLQ-C30 and the FACIT System^16^. Additional symptom items are identified through qualitative interviews (concept elicitation/cognitive interviews) of patients with the targeted disease, and the resulting instrument is validated in alignment with FDA guidance on the development of PRO instruments for use in regulatory evaluation and for labeling claims^3^. With this approach, the new instruments are offered as approximating the FDA’s intentions for the use of PROs as fit-for-purpose outcome measures for clinical assessment^4^.

The MDASI-MPM was developed with reference to the guidance described above. During the qualitative interviews, 6 additional MPM-specific symptom items (coughing, muscle weakness, feeling of malaise, chest heaviness or tightness, eye problems, and trouble with balance or falling) were identified; complete details of the qualitative phase of MDASI-MPM development are reported elsewhere^15^. The MDASI-MPM’s psychometric properties were evaluated in an open-label, blinded, randomized, multicenter Phase IIB trial in patients with MPM (described below)^13^; the instrument’s stability, reliability, and sensitivity to and responsiveness to changes in disease status were found to be excellent^12^.

### MDASI-MPM composite symptom score (CSS) development

The final component of the MDASI-MPM’s development was to generate the MPM-specific CSS^15^. The process for developing the CSS included (1) formation of an independent review committee (IRC) of experts in measure development and regulatory considerations; (2) development of the statistical analysis plan for the CSS; (3) development of a plan to blind and pool data from the Phase IIB trial to shield the PRO development team from treatment-specific information; (4) longitudinal examination of the performance of the candidate CSS items based on data from the Phase IIB trial; and (5) IRC review and approval of the final CSS and recommendations for a meaningful change in CSS score were proposed.

The Phase IIB trial and subsequent development of the CSS were conducted under Bayer HealthCare AG Integrated Clinical Study Protocol No. BAY 94-9343/15743^17^, V1.0 dated August 17, 2015 (ClinicalTrials.gov Identifier #NCT02610140). This multisite, multinational study was approved by the institutional review boards or independent ethics committees of more than 60 participating sites (see Supplementary Table S1)^13^. Informed consent was obtained from all participants. This research was performed in accordance with the Declaration of Helsinki and relevant guidelines and regulations.

#### Independent review committee

In order to provide an independent and unbiased review of symptom items to be included in the CSS, the sponsor commissioned an IRC comprising PRO clinical trial and statistical experts (AB, ACD, JAS, TS). The IRC was convened by an outside contract research organization, Clinical Outcomes Solutions (www.clinoutsolutions.com), which coordinated the IRC’s virtual meetings and summarized recommendations. The IRC was chaired by Tara Symonds, Chief Science Officer of Clinical Outcomes Solutions.

#### CSS statistical analysis plan

A statistical analysis plan for developing the CSS (distinct from the statistical analysis plan in the Phase IIB study protocol^13^) was developed and revised with guidance from the IRC at its first meeting. In accordance with this plan, longitudinal symptom data from the MDASI-MPM psychometric validation study^12^ were summarized for the IRC. To determine which elements best captured the prevalence and severity of MPM, the most severe symptoms from that study at baseline and during treatment were described, both by mean severity and by the percentage of patients reporting a symptom as moderate or severe (represented by a score of ≥ 5 or ≥ 7 on that item, respectively). The identified symptoms then formed a pool of candidate items to be reviewed for inclusion in the CSS.

The ≥ 5 cutpoint for individual symptoms came from studies showing that “pain at its worst” is related to greater interference with function when rated ≥ 5 by cancer patients^9,18,19^. The choice of ≥ 7 to delineate a severe symptom was based on work by Serlin et al.^18^ showing that a cutpoint of 7 separates moderate from severe pain and Mendoza et al.^20^ showing that a cutpoint of 7 optimally differentiates between moderate and severe fatigue. A rating of ≥ 7 has been used in routine clinical practice to define severe pain and fatigue^21^ and in a large multicenter cooperative study to describe symptom prevalence^22^. (Other methods for determining cutpoints have been described^23^).

As recommended by the IRC, data for potential candidate items were regressed on the MDASI-MPM interference items to identify which symptoms predicted the instrument’s total symptom interference score—an approach we have used previously to categorize pain as mild, moderate, or severe on the 0–10 scale^18,23^. A backward regression and a stepwise regression were conducted on the symptom items at baseline and during treatment. Effect sizes were calculated for each item by comparing ratings at baseline and the safety follow-up, to determine how the candidate items changed over time in the entire sample. After reviewing these initial analyses, the IRC considered whether to exclude certain items from further consideration for the CSS on the basis of their low severity over time or ambiguity of meaning.

Once the candidate CSS items were determined, we used Cronbach coefficient alphas to estimate the internal consistency reliability of the CSS at baseline, on Day 1 of treatment Cycle 3, and at the safety follow-up. We examined test–retest reliability from assessments made between Cycle 2 Day 1 and Cycle 3 Day 1 (when we expected minimal changes), based on intraclass correlations of 0.70 or higher. To assess convergent validity, we correlated the CSS with the Lung Cancer Symptom Scale–Mesothelioma (LCSS-Meso), a valid and reliable QOL measure designed for patients with non-small cell lung cancer and modified for use in patients with MPM^24–27^. To evaluate the relationship between the CSS and the parent scale, we correlated the CSS with the full MDASI-MPM.

To evaluate known-group validity, we used independent-sample *t*-tests with Eastern Cooperative Oncology Group performance status (ECOG PS), a physician-rated measure of functional ability^28^, as the grouping variable. The ECOG PS ratings range from 0 (fully active; able to carry on all pre-disease performance without restriction) to 4 (completely disabled; cannot perform self-care; totally confined to bed or chair)^28^. We calculated CSS effect sizes between patients with fully active (ECOG PS = 0) versus restricted active (ECOG PS = 1) performance status. To establish sensitivity, we assessed whether the CSS could detect worsening of symptoms among patients with deteriorating performance status (as a clinical estimate of worsening disease status).

#### Phase IIB trial

The MDASI-MPM validation data used to derive the candidate CSS items and to evaluate the psychometric properties of various combinations of these items came from a randomized, open-label, active-controlled, 2-arm, multicenter Phase IIB trial evaluating the safety and efficacy of anetumab ravtansine (BAY 94-9343) versus vinorelbine (2:1)^13^ The MDASI-MPM was included in the trial in accordance with recommendations from the FDA in its PRO guidance^3^. Of the 248 randomized participants in that trial, 239 had nonmissing MDASI-MPM data and were included in the CSS development study. In the final study report for this trial^13^, no statistically significant between-group differences in MDASI-MPM outcomes were found.

Other measures used in the Phase IIB trial include the LCSS-Meso and ECOG PS. We also included the LCSS-Meso as a second measure of MPM-related QOL, using ratings on its single global QOL item as an anchor in the CSS validation. A no more than ± 9-point change on the LCSS-Meso QOL item has been reported as identifying no to minimal change (i.e., a no-change group)^29^. This item also asks patients to rate their overall QOL over the past week on a 100-mm visual analog scale. For this trial, ECOG PS was collected at each clinic visit and was used as a measure of disease severity.

The MDASI-MPM was completed at baseline, on Days 1 and 15 of each cycle up to 3 cycles, and on Day 1 of Cycles 4, 5, and 6. The LCSS-Meso was administered at baseline and on Day 1 of up to 6 treatment cycles; ECOG PS was assessed at baseline and at Day 1 of each cycle up to 6 cycles. Patients also completed the MDASI-MPM and LCSS-Meso at a safety follow-up, when most patients had disease progression, allowing for additional sensitivity estimates.

#### Blinding and pooling of data

All analyses used for developing the CSS were conducted using blinded, pooled trial data. Although the Phase IIB study was open-label and the academic organization was a study site, treatment-related data remained with the sponsor’s trial team and were kept from the IRC and the academic organization’s PRO experts and statistical team. Data from the Phase IIB study were stripped of treatment-identifying information immediately after database lock for the study’s primary analysis, and only this blinded dataset was sent to the statistical team developing the CSS.

## Results

### First IRC meeting

Selection of the initial candidate symptom items for the CSS was based on the psychometric analysis of the full dataset in the MDASI-MPM validation study^12^. The goal was to identify which MDASI-MPM symptom items best represented prevalence and severity (i.e., the percentage of patients reporting a symptom as moderate or severe, represented by a score of ≥ 5 or ≥ 7 on that item, respectively) before and during the trial. See Supplementary Tables S2 and S3 for select timepoints and Supplementary Figs. S1 and S2 for all symptoms. Backward and stepwise regression methods were used to regress the candidate items on the 2 MDASI-MPM interference subscales (mood-related and activity-related) to identify which symptoms predicted the instrument’s total MDASI symptom interference score at baseline and during treatment. Moderate to large standardized regression coefficient values (range 0.16–0.47) guided the selection of candidate symptom items for the composite symptom score. Effect sizes were calculated for each item by comparing ratings at baseline and at the safety follow-up, to determine how the candidate items changed over time for the entire sample. With the exception of coughing, which remained stable, effects sizes ranged from 0.18 to 0.60.

On the basis of the initial data review and discussion, the IRC excluded several MDASI-MPM items from further consideration for the CSS, including nausea, vomiting, sadness, distress, numbness, eye problems, difficulty remembering, and lack of appetite, as their severity over time in the Phase IIB trial was low. The remaining 8 symptoms—the MDASI core items pain, fatigue, and shortness of breath and the MPM-specific items malaise, muscle weakness, coughing, trouble with balance, and chest heaviness or tightness—were carried forward by the IRC as potential CSS candidate items.

### Second IRC meeting

At its second meeting, the IRC discussed various CSS solutions (different combinations of the 8 symptom items identified at the first IRC meeting) and the combination of items that it would want to see tested for the finalization meeting. In evaluating the candidate CSS items, the IRC and the academic organization’s investigators were blinded to the treatment groups.

Although there was agreement on most of the 8 items chosen from the data-driven approach, the IRC recommended that that malaise be removed because it might be ambiguous for some patients to interpret. Trouble with balance and chest heaviness or tightness were removed because of low prevalence. The removal of MDASI-MPM items was therefore based on both qualitative (patient interview) and quantitative (psychometric) data and the clinical experience of IRC members.

### Third IRC meeting

During the third meeting, the IRC recommended that the final CSS include pain, fatigue, shortness of breath, muscle weakness, and coughing, on the basis of the analyses comparing all candidate CSS items. The IRC also discussed what might constitute a clinically meaningful change in the CSS, as a starting point for refining evolving methods of interpreting what is meaningful to patients. Converging data from distribution and anchor-based analyses indicated that a 1-point change in the CSS was minimally important. The IRC final report suggested considering a more conservative 2-point change for regulatory use, and this 2-point change was used in the analysis of the Phase IIB study.

### Psychometric properties of the final CSS

#### Reliability

Good internal consistency was seen for the chosen CSS solution: Cronbach alphas were 0.82 (95% CI 0.78–0.86) at baseline, 0.84 (95% CI 0.79 − 0.87) at Cycle 3 Day 1, and 0.80 (95% CI 0.73–0.86) at the safety follow-up. Test–retest reliability was assessed on a subset of patients by selecting timepoints when patients were clinically stable (Day 1 of Cycles 2 and 3). In addition, patients should have had minimal change in the QOL item of the LCSS-Meso at those timepoints (i.e., no more than a ± 9-point change on the LCSS-Meso QOL item between Cycles 2 and 3). Results (n = 82) showed that the intraclass correlation (0.84, 95% CI 0.78–0.88) was in line with psychometric recommendations for the proposed CSS^4^. See Supplementary Table S4 for item-item correlations.

#### Validity

The selected CSS items correlated with the LCSS-Meso (range 0.79–0.87), suggesting good concurrent validity (Table 1). As expected, the selected CSS also correlated with the full MDASI-MPM symptom items (range 0.92–0.94). The assessment of known-group validity was based on comparisons of fully active (ECOG PS = 0) and restricted active (ECOG PS = 1) groups. Significant differences in the CSS between the 2 groups were seen up to Cycle 3 Day 1 (all* P* < 0.05, 3 assessment timepoints), supporting the known-group validity of the CSS (Table 2).

**Table 1:** Concurrent validity by correlation of the CSS^a^ with the full MDASI-MPM and LCSS-Meso at various timepoints.

	Sample size	MDASI-MPM	LCSS-Meso
Baseline	240	0.92	0.79
Cycle 2 Day 1	197	0.92	0.84
Cycle 3 Day 1	161	0.93	0.84
Cycle 4 Day 1	131	0.93	0.85
Cycle 5 Day 1	110	0.94	0.87
Safety follow-up	96	0.93	0.84

^a^The arithmetic average of 5 MDASI-MPM symptom items (pain, fatigue, shortness of breath, coughing, and muscle weakness).

*CSS* MDASI-MPM composite symptom score, *LCSS-Meso* Lung Cancer Symptom Scale–Mesothelioma, *MDASI-MPM* MD Anderson Symptom Inventory for Malignant Pleural Mesothelioma.

**Table 2:** Known-group validity for the CSS^a^ by ECOG PS at various timepoints.

	Sample size	Fully active ECOG PS = 0^b^	Restricted active ECOG PS = 1^b^	Difference	95% CI for the difference
Baseline	239	2.6 (1.9)	3.6 (2.1)	− 1.0	− 1.6 to − 0.5
Cycle 2 Day 1	201	2.4 (1.7)	3.2 (2.0)	− 0.8	− 1.4 to − 0.2
Cycle 3 Day 1	160	2.3 (1.6)	3.4 (2.0)	− 1.1	− 1.7 to − 0.5
Cycle 4 Day 1	128	2.5 (1.9)	3.1 (2.1)	− 0.6	− 1.3 to 0.1
Cycle 5 Day 1	106	2.7 (1.9)	3.1 (2.0)	− 0.4	− 1.1 to 0.4
Safety follow-up	69	3.6 (1.5)	3.5 (2.1)	0.1	− 1.3 to 1.4

^a^The arithmetic average of the 5 MDASI-MPM CSS symptom items (pain, fatigue, shortness of breath, coughing, and muscle weakness).

*CSS* MDASI-MPM composite symptom score, *ECOG PS* Eastern Cooperative Oncology Group performance status, *MDASI-MPM* MD Anderson Symptom Inventory for Malignant Pleural Mesothelioma.

^b^ECOG PS = 0 indicates fully active; ECOG PS = 1 indicates restricted active. The CSS differed significantly up to Day 1 of Cycle 3 between patients who were fully active versus those who were restricted active.

Exploratory factor analysis using principal axis factoring showed a consistent single-factor solution explaining most of the common variance (range 55–61%) across all trial assessment points (Supplementary Table S5). This result suggests that the CSS items measure a common underlying construct of disease-related symptom burden.

#### Sensitivity

**Figure 1:**
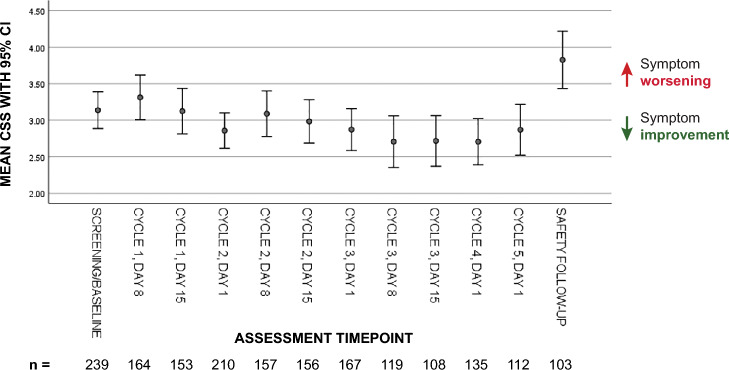
Mean severity and variability of the CSS over time. The significant increase at follow-up supports the notion that as the disease progresses, patients report worsening symptoms. CSS: MD Anderson Symptom Inventory for Malignant Pleural Mesothelioma composite symptom score.

Sensitivity to symptom change with disease progression was assessed by using Cohen’s *d* to calculate the effect size of change in mean CSS between baseline and the safety follow-up (n = 50). At baseline, the mean CSS was 3.3 ± 2.1 on the 0–10 scale; by the safety follow-up, the mean CSS had worsened to 4.5 ± 2.3, for a mean difference of − 1.2 points (95% CI − 1.90 to − 0.54; *P* = 0.001) and an effect size of 0.57 (Fig. 1).

### Proposed meaningful change in CSS

To estimate the meaningful change in the CSS for symptom worsening, we used ECOG PS as the anchor and stratified participants into 2 groups on the basis of change in ECOG PS between baseline and the safety follow-up. Because ECOG PS improved in only 3 patients, we combined the improved group and the no-change group (n = 42), and compared this combined group with the declining ECOG PS group (n = 50). Both the anchor-based and distribution-based results (Table 3) suggested that a 1-point increase in the CSS indicates meaningful worsening of symptoms.

**Table 3:** Meaningful change estimation for the CSS^a^.

Based on:	Anchor-based estimate^b^		Distribution-based estimate	
Change in ECOG PS^c^	Improved or no change (n = 45)	0.65 ± 2.09	One-half SD	1.12
			One-third SD	0.75
	Declining (n = 50)	1.22 ± 2.39	SEM	0.95
Change in the LCSS-Meso QOL item^d^	Improved (n = 61)	− 1.05 ± 1.96	One-half SD	0.99
	No change (n = 70)	0.16 ± 1.46	One-third SD	0.66
	Declining (n = 52)	0.65 ± 1.30	SEM	0.83

^a^The arithmetic average of 5 MDASI-MPM symptom items (pain, fatigue, shortness of breath, coughing, and muscle weakness).

*CSS* MDASI-MPM composite symptom score, *ECOG PS* Eastern Cooperative Oncology Group performance status, *LCSS-Meso* Lung Cancer Symptom Scale–Mesothelioma, *MDASI-MPM* MD Anderson Symptom Inventory for Malignant Pleural Mesothelioma, *QOL* quality of life, *SEM* standard error of the mean.

^b^Positive scores indicate that symptoms worsened (the CSS was higher than it was at baseline). Negative scores indicate that symptoms improved (the CSS was lower than it was at baseline).

^c^Change from baseline to the safety follow-up, (n = 95).

^d^Change from baseline to Day 1 of Cycle 2. A change no greater than ± 9 indicates no change.

For estimating meaningful change in the CSS for symptom improvement, we used the LCSS-Meso QOL item as the anchor and a no more than ± 9-point change to indicate a no-change group. We stratified participants into 3 groups, this time on the basis of change in the LCSS-Meso QOL item between baseline and Day 1 of Cycle 2: improved (n = 61), no change (n = 70), and declining (n = 52). Both the anchor-based and distribution-based results (Table 3) suggested that a 1-point change in the CSS indicates meaningful improvement in symptoms.

**Figure 2:**
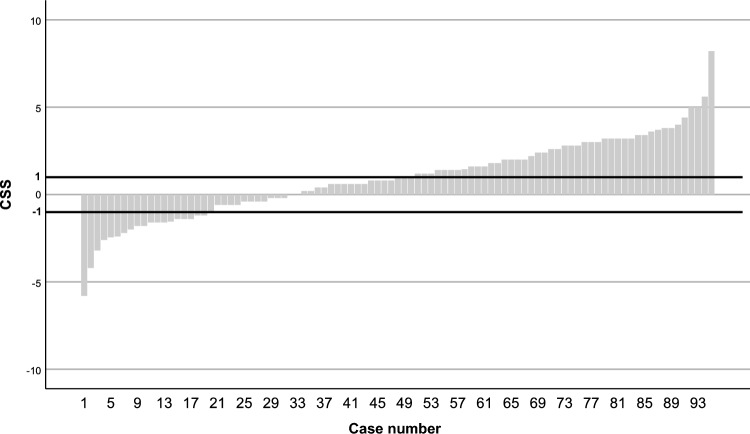
Waterfall plot showing the change in the CSS for each patient from baseline to safety follow-up. Positive scores indicate that symptoms got worse (the CSS increased). Negative scores indicate that symptoms improved (the CSS decreased). Given a minimally important difference of 1 (heavy lines at 1 and − 1), the plot shows that 21% of patients had symptom improvement while 47% had symptom worsening for the 95 patients who completed the MDASI-MPM at both baseline and the safety follow-up. CSS: MDASI-MPM composite symptom score; MDASI-MPM: MD Anderson Symptom Inventory for Malignant Pleural Mesothelioma.

With a 1-point change in the CSS as indicative of meaningful change over time compared with baseline, approximately 47% of patients showed worsening symptoms as measured by the CSS, and approximately 21% showed improvement (Fig. 2).

## Discussion

This report describes a method for developing and evaluating a composite symptom score (CSS) to represent disease-related symptom burden as a single variable in clinical trials targeting malignant pleural mesothelioma. The CSS was derived from items of the MDASI-MPM, which was developed and validated in a Phase IIB clinical trial as a component of an ongoing collaborative program^12,13^. The CSS is a subset of MDASI-MPM symptom items that summarizes change in disease-related symptom burden during and after a trial. Disease-related symptoms are those that are often present before treatment begins, as described for head and neck cancer^30^ and lung cancer^31^. The CSS takes 1–5 min to complete. Its limited number of symptoms and simple format make it easy administer electronically from outside of the clinic—for example, as an ePRO on a smartphone. Because the CSS can track change at the individual patient level, it can be used in a responder analysis, providing an additional patient-level perspective on a treatment’s impact on trial participants. The substantial overlap between the CSS and the full set of symptom items in this study supports less-frequent administration of the full scale—possibly only at major milestones, such as for staging, imaging, or protocol-specified clinic visits.

The method used to develop the CSS included input from an external independent review committee with expertise in the clinical application of PROs in clinical trials and regulatory experience. On the basis of sequential analyses of blinded, pooled longitudinal data from the Phase IIB trial, the IRC suggested methods for selecting CSS items from among the symptoms rated by patients as most severe at baseline, during the trial, and at the safety follow-up, when most patients were experiencing disease progression. The recommended final CSS included pain, fatigue (tiredness), shortness of breath, muscle weakness, and coughing. Muscle weakness and coughing emerged from the qualitative interviews of MPM patients^15^; the remaining CSS items were core MDASI symptoms^14^.

The psychometric properties of the CSS were examined using pooled, blinded data from the Phase IIB trial. The CSS was found to have good internal consistency and test–retest reliability. Principal component analysis conducted at 7 assessment timepoints strongly supported the unidimensional construct of symptom burden for the CSS. As expected, the CSS was also highly correlated (0.92–0.94) with the full MDASI-MPM symptom set from which it was derived and with the LCSS-Meso (0.79–0.87). The CSS was sensitive in detecting change—in this case, worsening symptom burden between baseline and the safety follow-up due to disease progression.

To the extent possible, regulatory agencies wish to evaluate disease-related symptom change as distinct from symptomatic change due to treatment toxicities^1^. Accordingly, the FDA recommends that, in PRO data submitted for review, disease-related symptoms should be separated from the toxicities of therapies (symptomatic adverse events)^1^. This is an active area of discussion in oncology trial design, and some symptoms can be both disease-related and treatment-related. Tracking change in symptom severity and daily functioning from pretreatment baseline to end of trial has been suggested as a way to approach this issue.

In its June 2021 draft guidance^4^, the FDA recommended that a core set of patient-reported symptoms representing disease and functional impacts as well as symptomatic toxicities be included in cancer clinical trials. They also proposed that disease-related symptoms can be measured individually or within a symptom score with other disease-related symptoms relevant to the cancer under study, and that a multisymptom measure that asks patients to rate each symptom at its worst during a specified time interval could be used.

The CSS is being proposed as an efficacy outcome representing disease-related symptom burden; it is not intended as a measure of symptomatic treatment toxicities. This is in contrast to the National Cancer Institute's Patient-Reported Outcomes Version of the Common Terminology Criteria for Adverse Events (PRO-CTCAE) measurement system, whose intended use is to describe the overall safety and tolerability of a therapeutic compound, regimen, or device^6^. The PRO-CTCAE was created primarily to assess symptomatic treatment toxicities, not to evaluate the potential modification of disease-related symptoms characteristic of a specific patient sample^32^.

The use of a composite score as a co-primary or secondary endpoint is not without precedent. The FDA approved the inclusion of a composite symptom score in labeling claims for ruxolitinib for the treatment of myelofibrosis^10^. This process employed a composite “total symptom score” comprising 6 symptoms items mutually agreed upon by the drug sponsor and the FDA as characterizing myelofibrosis^11,33^. The label presents the percentage of patients who reported a 50% reduction in this total symptom score^11,34^. The Average Symptom Burden Index (ASBI) from the LCSS-Meso has been used to measure symptom burden as a construct in non-small lung cancer trials and to demonstrate treatment equivalence or, in some cases, superiority in symptom control^35,36^. The ASBI averages the ratings of 5 symptoms (pain, fatigue, shortness of breath, coughing, and lack of appetite). The LCSS-Meso and the CSS differ in recall periods (24 h for the CSS, 1 week for the LCSS-Meso) and response options (numeric for the CSS, visual analog for the LCSS-Meso).

The derivation of the CSS also parallels the development of the FDA-qualified Non-Small Cell Lung Cancer Symptom Assessment Questionnaire (NSCLC-SAQ)^37,38^, a collaborative effort by a consortium of pharmaceutical company outcomes experts, academic investigators, and regulatory agencies in accordance with FDA guidance on clinical outcome assessment qualification^39^. Because we had access to longitudinal Phase IIB trial data, we were able to examine change in the CSS over time.

Of note, the sponsor interacted with our group to develop the trial, including seeking input from investigators who had experience using the target drug in Phase I/II studies to learn how these site investigators expected the drug to affect disease-related symptom burden. Moreover, the sponsor and the academic team presented plans for PRO instrument acceptance as a clinical outcome assessment in meetings with the FDA, per the FDA’s suggestion of early review of instruments and assessment schedules during instrument development.

A major limitation of this study is the rapid progression of disease in our sample, restricting the determination of symptom status associated with disease improvement. The data used to develop the CSS were derived primarily from patients with stage III or IV epithelioid MPM. Using the CSS in trials with earlier-stage disease would require recalibration of the symptom items for patients with less disease burden. Of note, the 2 agents investigated in the Phase IIB trial (anetumab ravtansine versus vinorelbine) exhibited limited clinical activity. A second limitation was the lack of blinding for both patients and trial site investigators. Nonetheless, the PRO academic investigators and the IRC were blinded as to treatment allocation. Finally, MPM is a cancer that has been a focal point of litigation and substantial financial settlements to patients, possibly creating a bias toward inflating symptom burden reporting. In such instances, PRO assessments, including the CSS, may not be used as designed. Because litigation status was missing from the current dataset, we were unable to investigate this possibility.

## Conclusion

We developed a CSS for the MDASI-MPM that was derived from both qualitative and quantitative data and refined on the basis of clinical opinion and psychometric analysis. Our procedures were informed by FDA guidance on the development of fit-for-purpose, PRO-based assessment instruments^3,4^. Convening a blinded independent review committee (the IRC) was an important feature in the development of the CSS, and having access to longitudinal Phase IIB trial data was invaluable for evaluating the psychometric properties of the composite score. This approach can be used to develop multi-item composite scores for other cancers (especially rapidly progressive disease) and treatments.

Various options are emerging for the treatment of relapsed epithelioid MPM—a previously unmet medical need. Using a brief, highly targeted assessment like the CSS may help to differentiate among novel treatments and to identify those that not only show efficacy but also reduce MPM-related symptom burden more effectively, thus leading to better treatment options and better quality of life for patients with MPM.

### Supplementary Information

Supplementary Information.

## Data Availability

Data are available on request from the corresponding author. Data are not publicly available due to privacy restrictions.
