# Supplementary material for: Developing a fit-for-purpose composite symptom score as a symptom burden endpoint for clinical trials in patients with malignant pleural mesothelioma
Source: Sci Rep. 2024 Jun 27;14:14839. doi: 10.1038/s41598-024-62307-5 (PMC11211485; doi:10.1038/s41598-024-62307-5)
Supplement: Supplementary file 1 — Supplementary Information. [file 41598_2024_62307_MOESM1_ESM.pdf]

SUPPLEMENTARY INFORMATION

Developing a fit-for-purpose composite symptom score as a symptom burden endpoint for clinical trials in patients with malignant pleural mesothelioma

Charles S. Cleeland, PhD; Karen N. Keating, MBA, RPT; Brian Cuffel, PhD; Cem Elbi, MD, PhD; Jonathan M. Siegel, MSc; Christoph Gerlinger, PhD; Tara Symonds, PhD; Jeff A. Sloan, PhD; Amylou C. Dueck, PhD; Andrew Bottomley, PhD; Xin Shelley Wang, MD, MPH; Loretta A. Williams, PhD, APRN; Tito R. Mendoza, PhD, MSc, MEd

Supplementary Table S1. Phase IIB study sites and investigators.

Country	Investigator	Institution	Number of patients randomized
Australia	Ross Jennens	Epworth HealthCare	8
	Nick Pavlakis	Northern Cancer Institute	1
	Anna Nowak	Sir Charles Gairdner Hospital	5
Belgium	Koen Deschepper	AZ Nikolaas Campus Sint-Niklaas	1
	Anne Sibille	CHU de Liège	2
	Thierry Pieters	CU Saint-Luc/UZ St Luc	2
	Jan Van Meerbeeck	UZ Antwerpen	5
	Veerle Surmont	UZ Gent	2
	Kris Nackaerts	UZ Leuven Gasthuisberg	9
Canada	Desiree Hao	Tom Baker Cancer Centre	3
Finland	Aija Knuuttila	HUS, Meilahden sairaala	4
	Taneli Saariaho	Turun yliopistollinen keskussairaala, kantasairaala	3
	Antti Jekunen	Vaasan keskussairaala	2
France	Radj Gervais	Centre François Baclesse - CLCC - Caen	1
	Pierre-Jean Souquet	Centre Hospitalier Lyon Sud – Pierre Bénite	3
	Arnaud Scherpereel	Hôpital Albert Calmette	1
	Gérard Zalcman	Hôpital Bichat - Paris	2
	Laurent Greillier	Hôpital du Nord - Marseille	1
	Jacques Cadranel	Hôpital Tenon - Paris	1
	Sylvestre Le Moulec	Institut Bergonié - CRLCC - Bordeaux	3
Italy	Silvia Novello	A.O.U. San Luigi Gonzaga	8
	Michele Maio	A.O.U. Senese	8
	Diego Cortinovis	ASST Monza (since 01 Jan 2016)	4
	Giovanni Luca Ceresoli	Cliniche Gavazzeni SpA	14
	Marina Chiara Garassino	Fondazione IRCCS Istituto Nazionale dei Tumori di Milano	3
	Alessandra Bearz	IRCCS Centro di Riferimento Oncologico - CRO	16
	Armando Santoro	IRCCS Istituto Clinico Humanitas - Humanitas Mirasole SpA	7
Netherlands	Joachim G.J.V. Aerts	Erasmus Medisch Centrum	12
	Jacobus A. Burgers	Nederlands Kanker Instituut	8
Poland	Piotr Serwatowski	Specjalistyczny Szpital im. prof. Alfreda Sokolowskiego	6
	Grzegorz Czyzewicz	Szpital Specjalistyczny im. Jana Pawla II	2
	Artur Drobniak	Szpital Uniwersytecki w Krakowie	1
	Jacek Jassem	Uniwersyteckie Centrum Kliniczne	1
Russia	Mikhail Dvorkin	Clinical Oncological Dispensary of Omsk Region	1

South Korea	DaeHo Lee	Asan Medical Center	1
	Se-Hoon Lee	Samsung Medical Center	1
Spain	Susana Cedrés	Ciutat Sanitària i Universitaria de la Vall d' Hebron	7
	Rosario García Campelo	Complejo Hospitalario Universitario A Coruña	2
	Bartomeu Massuti	Hospital General Universitario de Alicante	4
	Luis Paz Ares	Hospital Universitario 12 de Octubre	6
	José Manuel Trigo Pérez	Hospital Virgen de la Victoria	2
Turkey	Berna Oksuzoglu	Ankara Oncology Training and Research Hospital	2
	Ismail Kara	Cukurova Universitesi Tip Fakultesi Hastanesi	2
	Suayip Yalcin	Hacettepe Universitesi Tip Fakultesi	1
	Perran Yumuk	Marmara Universitesi Tip Fakultesi	3
United Kingdom	Nicola Steele	Beatson West of Scotland Cancer Centre	1
	Amy Roy	Derriford Hospital	3
	Alastair Greystoke	Freeman Hospital	9
	James Spicer	Guy's Hospital	11
	Riyaz Shah	Kent Oncology Centre	2
	Dean Fennell	Leicester Royal Infirmary	7
	Paul Taylor	Wythenshawe Hospital	11
United States	James Stevenson	Cleveland Clinic	1
	Jeffrey Crawford	Duke University Medical Center	1
	Dennis Slater	Eastern Connecticut Hematology/Oncology Associates	3
	Tawee Tanvetyanon	H. Lee Moffitt Cancer Center & Research Institute	4
	John Nemunaitis	Mary Crowley Medical Research Center	1
	Aaron Mansfield	Mayo Clinic - Rochester	1
	Raffit Hassan	National Institutes of Health	1
	Robert Ramirez	Ochsner Medical Center - New Orleans	3
	Grace Dy	Roswell Park Cancer Institute	2
	Marta Batus	Rush University Medical Center	2
	Lyudmila Bazhenova	University of California San Diego Moores Cancer Center	1
	Hedy Kindler	University of Chicago	3

Supplementary Table S2. Mean MD Anderson Symptom Inventory for malignant pleural mesothelioma (MDASI-MPM) symptom ratings at three timepoints* during the study.

Symptom	Baseline			Cycle 3 Day 1			Safety follow-up		
	N	Mean	SD	N	Mean	SD	N	Mean	SD
Fatigue†	236	4.1	2.6	166	3.8	2.5	104	5.0	2.5
Shortness of breath†	237	3.7	2.7	167	3.2	2.6	103	4.2	2.9
Pain†	237	3.0	2.6	167	2.6	2.3	104	3.7	2.7
Distress	238	2.8	2.7	166	2.5	2.5	104	3.0	2.6
Muscle weakness†	238	2.7	2.7	165	3.0	2.6	104	4.1	3.1
Malaise	238	2.7	2.6	165	2.8	2.3	103	3.3	2.7
Lack of appetite	238	2.6	2.9	166	2.4	2.6	104	3.0	2.6
Disturbed sleep	238	2.6	2.7	167	2.5	2.4	104	3.2	2.6
Drowsiness	239	2.5	2.6	165	2.5	2.2	104	3.3	2.5
Chest heaviness/tightness	236	2.3	2.4	165	2.1	2.2	103	2.8	2.6
Sadness	239	2.3	2.7	167	2.3	2.6	104	2.8	2.9
Coughing†	239	2.2	2.4	165	1.7	2.0	104	2.1	2.2
Dry mouth	236	2.1	2.6	167	2.0	2.2	104	2.5	2.4
Difficulty remembering	239	1.9	2.3	166	1.8	2.2	104	2.1	2.3
Numbness/tingling	239	1.6	2.4	167	2.3	2.6	104	3.4	3.1
Eye problems	236	1.1	1.9	165	1.5	2.0	104	1.6	2.1
Imbalance/falling	238	1.1	1.9	164	1.3	1.9	104	2.5	2.7
Nausea	236	1.0	1.9	167	1.0	1.6	101	1.2	1.8
Vomiting	237	0.5	1.6	166	0.5	1.3	104	0.8	1.5

* Patients were expected to be clinically stable between baseline and Cycle 3 Day 1 and to be experiencing disease progression by the time of the safety follow-up. These timepoints were used to investigate test-retest reliability and sensitivity, respectively.

† Selected CSS items.

Supplementary Table S3. Proportion of patients with moderate to severe and severe symptoms at three timepoints* during the study.

Symptom	Baseline			Cycle 3 Day 1			Safety follow-up		
	N	Moderate to severe %	Severe %	N	Moderate to severe %	Severe %	N	Moderate to severe %	Severe %
Pain	236	.31	.13	166	.16	.08	104	.38	.20
Fatigue (tiredness)	237	.47	.20	167	.39	.19	103	.56	.28
Nausea	237	.07	.03	167	.05	.02	104	.09	.01
Disturbed sleep	238	.26	.13	166	.23	.08	104	.32	.12
Distress	238	.26	.12	165	.19	.08	104	.29	.12
Shortness of breath	238	.39	.19	165	.29	.15	103	.45	.27
Difficulty remembering	238	.16	.04	166	.11	.07	104	.19	.05
Lack of appetite	238	.24	.14	167	.19	.08	104	.31	.12
Feeling drowsy	239	.23	.13	165	.18	.07	104	.34	.13
Dry mouth	236	.19	.09	165	.16	.05	103	.23	.08
Feeling sad	239	.22	.10	167	.20	.11	104	.27	.13
Vomiting	239	.03	.02	165	.02	.01	104	.05	.01
Numbness or tingling	236	.15	.07	167	.18	.10	104	.35	.22
Coughing	239	.20	.07	166	.10	.04	104	.16	.06
Feeling of malaise	239	.25	.10	167	.24	.08	104	.34	.16
Imbalance/falling	236	.07	.04	165	.08	.03	104	.21	.11
Chest heaviness or tightness	238	.19	.08	164	.13	.06	104	.27	.13
Muscle weakness	236	.24	.13	167	.27	.14	101	.43	.24
Eye problems	237	.08	.02	166	.10	.05	104	.12	.05

* Patients were expected to be clinically stable between baseline and Cycle 3 Day 1 and to be experiencing disease progression by the time of the safety follow-up. These timepoints were used to investigate test-retest reliability and sensitivity, respectively.

Supplementary Table S4. Bivariate correlation matrix for the five composite symptom score items at three timepoints* during the study.

	Shortness of Breath	Coughing	Muscle weakness	Pain
Baseline (n=236)				
Fatigue	.630	.393	.653	.603
Shortness of breath		.511	.567	.425
Coughing			.337	.254
Muscle weakness				.422
Cycle 3, Day 1 (n=166)				
Fatigue	.692	.499	.722	.574
Shortness of breath		.494	.517	.313
Coughing			.423	.257
Muscle weakness				.538
Safety follow up (n=103)				
Fatigue	.594	.394	.691	.600
Shortness of breath		.330	.466	.379
Coughing			.250	.299
Muscle weakness				.456

* Patients were expected to be clinically stable between baseline and Cycle 3 Day 1 and to be experiencing disease progression by the time of the safety follow-up. These timepoints were used to investigate test-retest reliability and sensitivity, respectively.

Supplementary Table S5. Factor matrix of the composite symptom score items and percent variance explained by the single factor at three timepoints* during the study.

	Baseline	Cycle 3 Day 1	Safety follow-up
Percent of variance explained	59%	61%	57%
**Symptom**	**Factor loadings**		
Fatigue (tiredness)	.870	.953	.949
Shortness of breath	.772	.780	.714
Muscle weakness	.730	.705	.642
Pain	.601	.570	.631
Coughing	.506	.552	.424

* Patients were expected to be clinically stable between baseline and Cycle 3 Day 1 and to be experiencing disease progression by the time of the safety follow-up. These timepoints were used to investigate test-retest reliability and sensitivity, respectively.

Supplementary Figure S1. Proportions of patients reporting moderate to severe levels over time of MDASI-MPM symptom items selected for inclusion in the CSS. CSS: composite symptom score; MDASI-MPM: MD Anderson Symptom Inventory for malignant pleural mesothelioma.

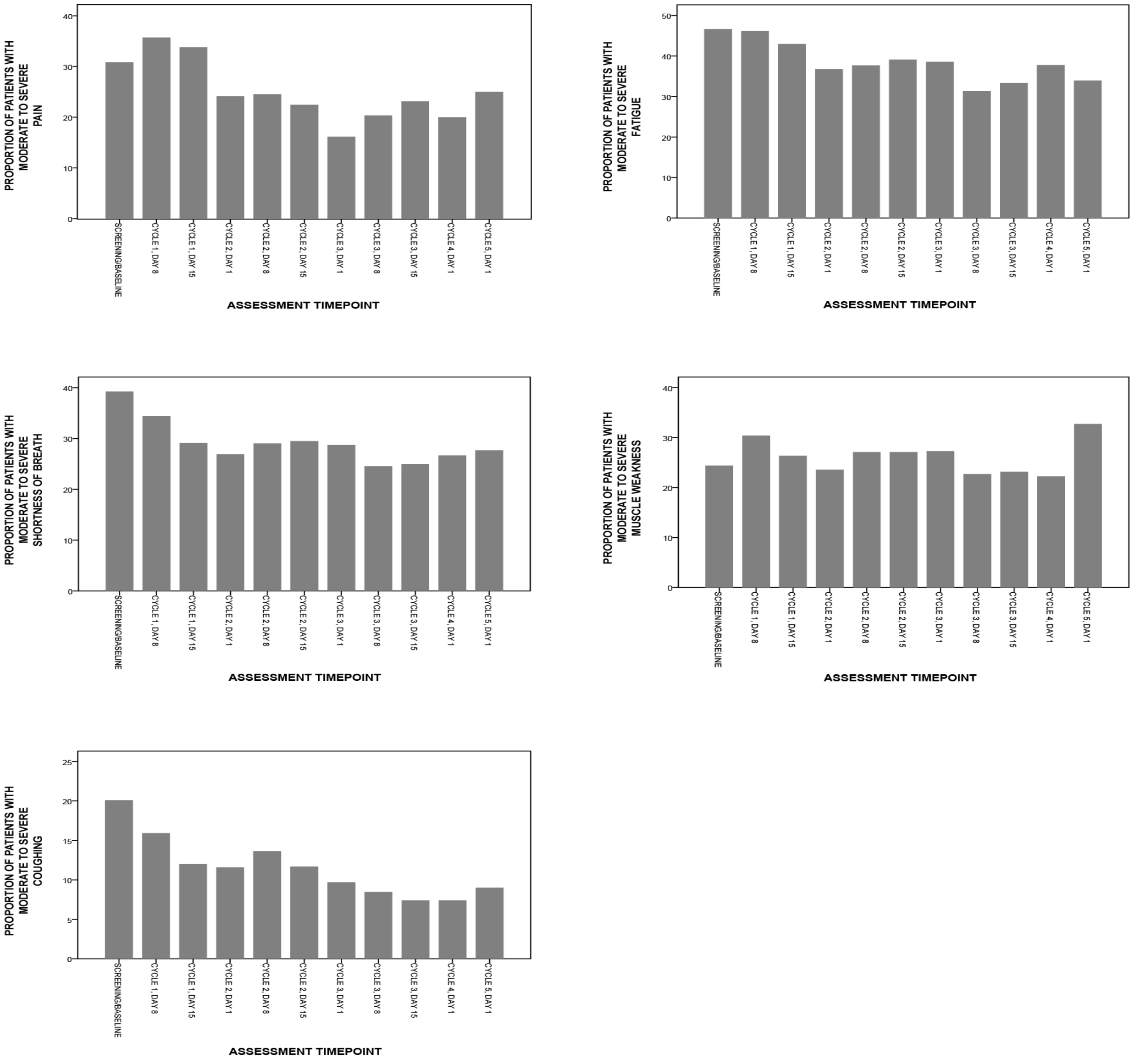

Supplementary Figure S2. Proportions of patients reporting moderate to severe levels over time of MDASI-MPM symptom items not selected for inclusion in the CSS. CSS: composite symptom score; MDASI-MPM: MD Anderson Symptom Inventory for malignant pleural mesothelioma.

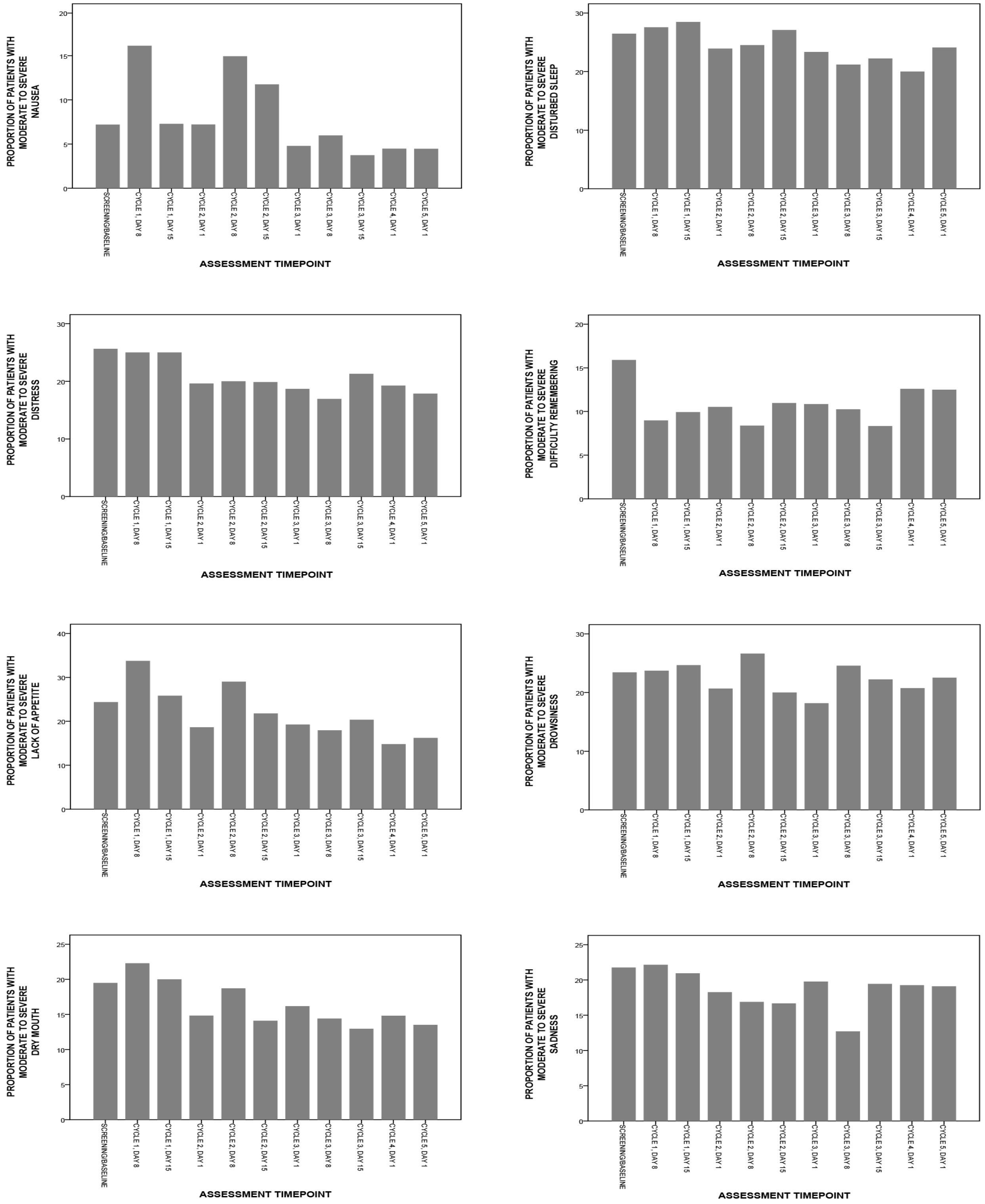

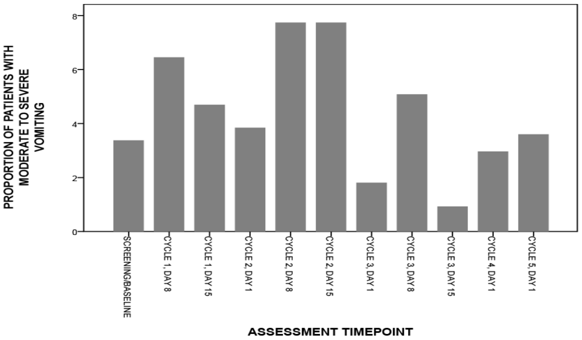
PROPORTION OF PATIENTS WITH MODERATE TO SEVERE VOMITING
0
2
4
6
8
SCREENING/BASELINE
CYCLE 1, DAY 8
CYCLE 1, DAY 15
CYCLE 2, DAY 1
CYCLE 2, DAY 8
CYCLE 2, DAY 15
CYCLE 3, DAY 1
CYCLE 3, DAY 8
CYCLE 3, DAY 15
CYCLE 4, DAY 1
CYCLE 5, DAY 1
ASSESSMENT TIMEPOINT

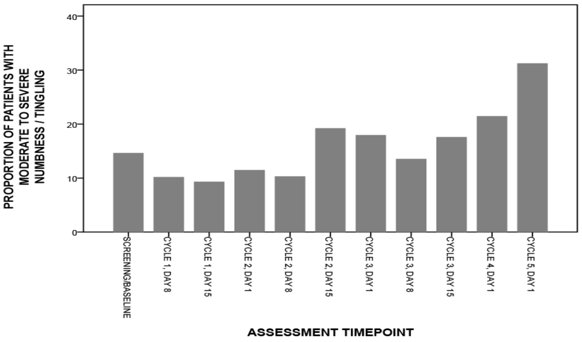
PROPORTION OF PATIENTS WITH MODERATE TO SEVERE NUMBNESS / TINGLING
0
10
20
30
40
SCREENING/BASELINE
CYCLE 1, DAY 8
CYCLE 1, DAY 15
CYCLE 2, DAY 1
CYCLE 2, DAY 8
CYCLE 2, DAY 15
CYCLE 3, DAY 1
CYCLE 3, DAY 8
CYCLE 3, DAY 15
CYCLE 4, DAY 1
CYCLE 5, DAY 1
ASSESSMENT TIMEPOINT

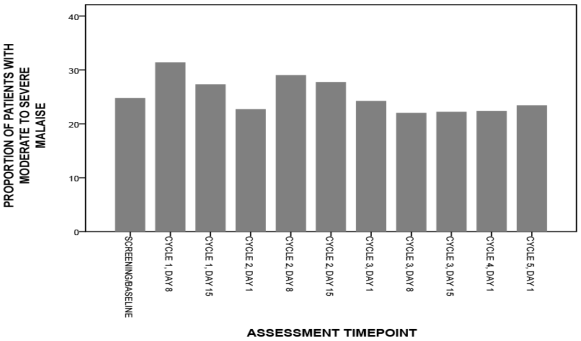
PROPORTION OF PATIENTS WITH MODERATE TO SEVERE MALAISE
0
10
20
30
40
SCREENING/BASELINE
CYCLE 1, DAY 8
CYCLE 1, DAY 15
CYCLE 2, DAY 1
CYCLE 2, DAY 8
CYCLE 2, DAY 15
CYCLE 3, DAY 1
CYCLE 3, DAY 8
CYCLE 3, DAY 15
CYCLE 4, DAY 1
CYCLE 5, DAY 1
ASSESSMENT TIMEPOINT

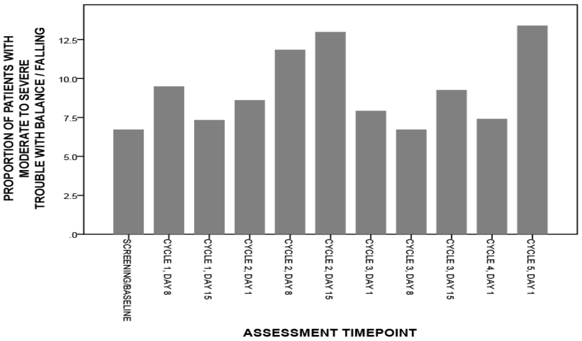
PROPORTION OF PATIENTS WITH MODERATE TO SEVERE TROUBLE WITH BALANCE / FALLING
.0
2.5
5.0
7.5
10.0
12.5
SCREENING/BASELINE
CYCLE 1, DAY 8
CYCLE 1, DAY 15
CYCLE 2, DAY 1
CYCLE 2, DAY 8
CYCLE 2, DAY 15
CYCLE 3, DAY 1
CYCLE 3, DAY 8
CYCLE 3, DAY 15
CYCLE 4, DAY 1
CYCLE 5, DAY 1
ASSESSMENT TIMEPOINT

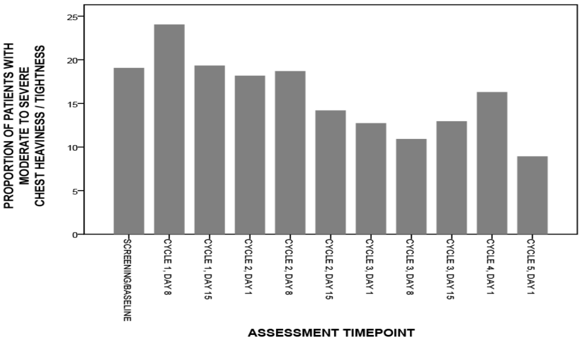
PROPORTION OF PATIENTS WITH MODERATE TO SEVERE CHEST HEAVINESS / TIGHTNESS
0
5
10
15
20
25
SCREENING/BASELINE
CYCLE 1, DAY 8
CYCLE 1, DAY 15
CYCLE 2, DAY 1
CYCLE 2, DAY 8
CYCLE 2, DAY 15
CYCLE 3, DAY 1
CYCLE 3, DAY 8
CYCLE 3, DAY 15
CYCLE 4, DAY 1
CYCLE 5, DAY 1
ASSESSMENT TIMEPOINT

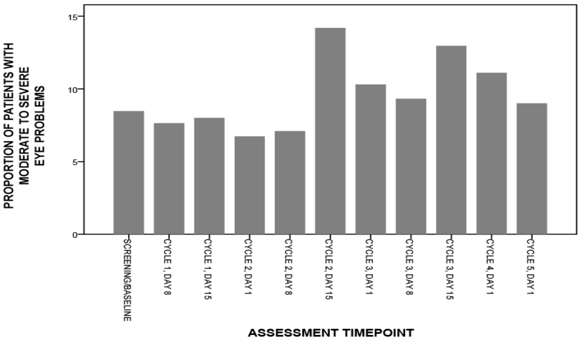
PROPORTION OF PATIENTS WITH MODERATE TO SEVERE EYE PROBLEMS
0
5
10
15
SCREENING/BASELINE
CYCLE 1, DAY 8
CYCLE 1, DAY 15
CYCLE 2, DAY 1
CYCLE 2, DAY 8
CYCLE 2, DAY 15
CYCLE 3, DAY 1
CYCLE 3, DAY 8
CYCLE 3, DAY 15
CYCLE 4, DAY 1
CYCLE 5, DAY 1
ASSESSMENT TIMEPOINT
